# Trends in the Degree of Control and Treatment of Cardiovascular Risk Factors in People With Type 2 Diabetes in a Primary Care Setting in Catalonia During 2007–2018

**DOI:** 10.3389/fendo.2021.810757

**Published:** 2022-01-10

**Authors:** Manel Mata-Cases, Bogdan Vlacho, Jordi Real, Ramon Puig-Treserra, Magdalena Bundó, Josep Franch-Nadal, Didac Mauricio

**Affiliations:** ^1^ Grup de Recerca Epidemiològica en Diabetis des de l'Atenció Primària (DAP-CAT), Unitat de Suport a la Recerca Barcelona, Fundació Institut Universitari per a la Recerca a l’Atenció Primària de Salut Jordi Gol i Gurina (IDIAPJGol), Barcelona, Spain; ^2^ Centre d’Atenció Primària La Mina, Gerència d’Àmbit d’Atenció Primària de Barcelona, Institut Català de la Salut, Barcelona, Spain; ^3^ Centro de Investigación Biomédica en Red de Diabetes y Enfermedades Metabólicas Asociadas, (CIBERDEM), Barcelona, Spain; ^4^ Centre d’Atenció Primària Ronda Prim, Gerència d’Àmbit d’Atenció Primària Metropolitana Nord de Barcelona, Institut Català de la Salut, Mataro, Spain; ^5^ Primary Health Care Center Raval Sud, Gerència d’Atenció Primaria, Institut Català de la Salut, Barcelona, Spain; ^6^ Department of Endocrinology & Nutrition, Hospital de la Santa Creu i Sant Pau, IIB Sant Pau, Spain; ^7^ Departament of Medicine, University of Vic, Central University of Catalonia, Vic, Spain

**Keywords:** Type 2 dabetes, antidiabetic drugs, glycemic concrol, epidemiology, observational study

## Abstract

**Objective:**

To assess the trends in cardiovascular risk factor control and drug therapy from 2007 to 2018 in subjects with type 2 diabetes mellitus (T2DM).

**Materials and Methods:**

Cross-sectional analysis using yearly clinical data and treatment obtained from the SIDIAP database. Patients aged ≥18 years with a diagnosis of T2DM seen in primary care in Catalonia, Spain.

**Results:**

The number of T2DM patients increased from 299,855 in 2007 to 394,266 in 2018. We also found an increasing prevalence of cardiovascular disease, heart failure, and chronic kidney disease (from 18.4 to 24.4%, from 4.5 to 7.3%, and from 20.2 to 31.3%, respectively). The achievement of glycemic targets (HbA1c<7%) scarcely changed (54.9% to 55.9%). Major improvements were seen in blood pressure (≤140/90 mmHg: from 55% to 71.8%), and in lipid control (low-density lipoprotein cholesterol <100 mg/dl: 33.4% to 48.4%), especially in people with established cardiovascular disease (48.8 to 69.7%). Simultaneous achievement of all three targets improved from 12.5% to 20.1% in the overall population and from 24.5% to 32.2% in those with cardiovascular disease but plateaued after 2013. There was an increase in the percentage of patients treated with any antidiabetic drug (70.1% to 81.0%), especially metformin (47.7% to 67.7%), and DPP4i (0 to 22.6%). The use of SGLT-2 and GLP-1ra increased over the years, but remained very low in 2018 (5.5% and 2.1% of subjects, respectively). There were also relevant increases in the use of statins (38.0% to 49.2%), renin-angiotensin system (RAS) drugs (52.5% to 57.2%), and beta-blockers (14.3% to 22.7%).

**Conclusions:**

During the 2007-2018 period, relevant improvements in blood pressure and lipid control occurred, especially in people with cardiovascular disease. Despite the increase in the use of antidiabetic and cardiovascular drugs, the proportion of patients in which the three objectives were simultaneously achieved is still insufficient and plateaued after 2013. The use of antidiabetic drugs with demonstrated cardio renal benefits (SGLT-2 and GLP-1ra) increased over the years, but their use remained quite low.

## Introduction

Type 2 diabetes mellitus (T2DM) is a global health problem due to its high worldwide prevalence, high cost of management, associated chronic complications, disability, and premature deaths (1). Tight glycemic, blood pressure, and lipid control lower the risk of diabetes-related complications and death, especially when attained concomitantly. Consequently, multifactorial risk-factor control forms the foundation of clinical care for patients with T2DM (2–7). There has been consensus regarding several major goals of diabetes treatment for over two decades: achieving treatment targets for hemoglobin A1c (HbA1c) levels, blood pressure (BP), and low-density lipoprotein cholesterol (LDL-C) levels, as well as promoting smoking cessation (2–7). Previous studies reported that achievement of the three diabetes treatment goals had improved from the late 1990s to 2018 in the United States (US) (8–10), mainly from 1999 to 2010 (8), but recent analyses suggest that progress may have stalled or reversed in later periods (9, 10). Furthermore, a study in the United Kingdom (UK) showed that control of cardiovascular risk factors (CVRFs) remained suboptimal among both sexes (11), while another study reported that CVRFs had worsened especially among the overweight and obese adults (12).

Several studies have been published showing changes in the prescription of antidiabetic drugs (13–18). In the UK, an observational study reported an increase in metformin prescriptions and a decrease in sulphonylureas prescriptions between 2000 and 2017 (13). A study from Austria between 2012 and 2018 reported that metformin (alone or in combination) was the most frequently prescribed drug, and its use increased over the years; the prescriptions of SGLT-2i and GLP-1ra also increased, while prescriptions of sulphonylureas decreased (14). Several studies showed similar trends in the prescription of antidiabetic drugs in the United States (US): metformin remains the dominantly prescribed drug, together with insulins and sulfonylureas, while increased use has been observed mainly for Dipeptidyl peptidase-4 inhibitors (DPP-4i) and Glucagon-like peptide-1 receptor agonist (GLP-1ra) (15–17). In Catalonia, using the SIDIAP (Sistema per el Desenvolupament de la Investigació en Atenció Primària) population database, our group reported that despite the increasing use of new antidiabetic drugs, no clinically relevant changes were observed in glycemic control from 2007 to 2013 (18).

The healthcare system in Catalonia (Spain) is public and universal, where the primary care centers provide first contact and continuing care for persons with any health concerns, and they are usually the principal place where T2DM is diagnosed and managed. The antidiabetic treatment is free of charge for retired and severely ill people, while active subjects pay just a small part of the cost of the drugs. Since 2006, a system of electronic medical records (EMR), called e-CAP, was fully implemented in primary care, which allowed the creation of the SIDIAP population database (19, 20). To date, it is not known whether there has been progress in the degree of control of the three primary objectives (HbA1c, BP, and LDL-C) in our primary care environment.

Due to important changes in the therapeutic guidelines, we hypothesized that there would be changes in the control of cardiovascular risk factors (CVRFs) and pattern of use of antidiabetic drugs in our T2DM population. Our study aimed to describe the degree of control and treatment pattern related to CVRFs during 2007-2018 in primary care centers in Catalonia (Spain).

## Materials and Methods

### Design and Settings

We obtained annual cross-sectional data from 2007 to 2018 using the primary health care SIDIAP database. This database includes secondary pseudo-anonymized routinely collected health data from subjects attended in the primary health care centers (PHCCs) of the leading health care provider in Catalonia (Spain), the Catalan Institute of Health (Institut Català de la Salut, ICS). The SIDIAP database is a well-recognized and valid database for the study of diabetes, including EMR, clinical and laboratory parameters, and medicine prescription and dispensation data. In 2018, the ICS managed 288 PHCCs that served 5,672,956 registered citizens, 75.2% of the Catalan population.

### Eligibility Criteria

We included all subjects 18 years or older with a diagnosis of T2DM in the database defined by the International Statistical Classification of Diseases and Related Health Problems (ICD-10) diagnostic codes (E11 or E14 and sub-codes). We excluded all subjects with diagnostic codes of other types of diabetes such as type 1, gestational, or other (E10, O24, E13, respectively).

### Study Variables

For each year evaluated, the “cut-off” date was defined as December 31st. We collected variables related to the social-demographic characteristics (age, sex, smoking status, and alcohol consumption) and duration of T2DM. Comorbidities, such as hypertension and hypercholesterolemia, were defined on the base of the specific diagnostic code and/or drug treatment for any of these conditions. Peripheral artery disease, cerebrovascular disease, heart failure, ischemic heart disease, peripheral neuropathy, retinopathy as well as composite variables for microvascular and macrovascular complications were defined by the diagnostic codes of these conditions. Chronic kidney disease (CKD) was defined by the specific diagnostic code and/or the combination of CKD-EPI glomerular filtration rate <60 ml/min/1,73m2 and/or an albumin/creatinine ratio >30mg/g. The closest measurement to the “cut-off” date for clinical variables (systolic and diastolic blood pressure and body mass index -BMI) and laboratory parameters (HbA1c, lipid profile, renal profile) were considered. Drug treatment dispensations during the whole year (antidiabetic, antithrombotic, antihypertensive, and lipid-lowering drugs) were collected. Six steps of antidiabetic treatment were considered: non-pharmacological therapy (no drugs), non-insulin antidiabetic drug (NIAD) monotherapy, NIAD double therapy, NIAD triple therapy, insulin alone and insulin in combination. The non-pharmacological therapy was defined if there were no records for dispensing antidiabetic drugs during the previous year. Monotherapy was defined for NIADs and insulin separately. Dual therapy was defined as a combination of two NIADs, while triple therapy was a combination of three NIADs. Insulin in combination included all subjects with insulin in combination with any NIAD.

### Statistical Methods

Descriptive analysis for study variables was done for each year of observation. We calculated mean and standard deviation for all quantitative variables, and frequencies and percentages for qualitative variables. We calculated the degree of glycemic, blood pressure, and lipid control and the use of antihypertensive, lipid-lowering, antithrombotic, and glucose-lowering therapies. According to local and international guidelines, glycemic control was defined at an HbA1c level below 7%; we also used an HbA1c threshold below 8%, which is the pay-for-performance goal at our institution (ICS). We used the mean of all available BP measurements (usually 3-4 readings) to estimate each patient’s systolic and diastolic BP. BP control was defined as a systolic/diastolic BP level equal or less than 140/90 mm Hg. Cholesterol control was defined as having an LDL-C level less than 100 mg/dL. We further defined a composite indicator using the three outcomes (HbA1c, BP, and LDL-C). LDL-C control and the combined indicator were analyzed globally and separately in people with and without cardiovascular disease (CVD). Additionally, we performed trend tests to analyze whether the reported changes were statistically significant. For the continuous variables, we applied one-factor ANOVA, where the factors were the different year periods we wanted to compare (2007-2018). For categorical variables, we used the “prop_trend_test” function of the R Package rstatix (version 0.7.0). This function performs chi-squared test to assess the trend in proportions. Data management and all analyses were performed using R statistical software, version 3.6.3. (2020/02/29).

### Institutional Review Board Statement

The studies involving human participants were reviewed and approved by Institutional Review Board (or Ethics Committee) of IDIAP Jordi Gol i Gurina Foundation (protocol code 21/111-P and date of approval 04/05/2021). Written informed consent for participation was not required for this study in accordance with the national legislation and the institutional requirements.

## Results

### Characteristics of the Subjects

Characteristics of the study subjects at each time-point of the study are presented in **Table 1**. During the 12 years of observation, the population identified with T2DM in our database increased from 299,855 in 2007 to 394,266 subjects in 2018. The mean age increased from 68.4 to 70.3 years, and the proportion of males was from 51.9% to 55%. Regarding toxic habits, smokers decreased from 18.1% in 2007 to 14.4% in 2018, and high-risk alcohol consumption decreased from 2.9% to 1.2%. An increase in the prevalence of cardiovascular disease, heart failure, and chronic kidney disease was observed (from 18.4 to 24.4%, from 4.5 to 7.3% and from 20.2 to 31.3%, respectively).

**Table 1:** Characteristics of the subjects during the study period.

	2007	2008	2009	2010	2011	2012	2013	2014	2015	2016	2017	2018	p-value

N	299855	318065	335771	355019	369600	384826	395470	402312	401175	404252	400209	394266	–
Age, mean (SD), years	68.4 (12.2)	68.6 (12.3)	68.8 (12.4)	68.9 (12.5)	69.1 (12.6)	69.3 (12.6)	69.5 (12.7)	69.7 (12.7)	69.9 (12.6)	70.1 (12.6)	70.2 (12.6)	70.3 (12.5)	<0.001*
Age >75 years, n (%)	98104 (32.7)	107607 (33.8)	115629 (34.4)	124675 (35.1)	132801 (35.9)	140657 (36.6)	144226 (36.5)	145462 (36.2)	147890 (36.9)	149950 (37.1)	148749 (37.2)	148385 (37.6)	<0.001**
Diabetes duration, (years)	5.72 (5.58)	6.17 (5.59)	6.56 (5.63)	6.93 (5.71)	7.32 (5.80)	7.68 (5.92)	8.04 (6.03)	8.46 (6.08)	8.89 (6.17)	9.33 (6.30)	9.76 (6.42)	10.2 (6.57)	<0.001*
Sex (male), n (%)	155534 (51.9)	166499 (52.3)	177329 (52.8)	188809 (53.2)	197641 (53.5)	206793 (53.7)	213202 (53.9)	217706 (54.1)	218271 (54.4)	220818 (54.6)	219289 (54.8)	216708 (55.0)	<0.001**
**Smoking habit, n (%)**													
No smoker	151906 (64.5)	169301 (64.3)	186097 (64.0)	212027 (64.7)	224377 (64.3)	233993 (63.7)	233957 (61.4)	230948 (59.2)	224317 (57.3)	221448 (55.9)	216008 (54.8)	209774 (53.8)	<0.001**
Ex-smoker	42554 (18.1)	46441 (17.6)	49119 (16.9)	50377 (15.4)	50347 (14.4)	51658 (14.1)	54518 (14.3)	56625 (14.5)	56756 (14.5)	57323 (14.5)	57457 (14.6)	56060 (14.4)	<0.001**
Current smoker	40926 (17.4)	47488 (18.0)	55656 (19.1)	65449 (20.0)	74050 (21.2)	81493 (22.2)	92541 (24.3)	102432 (26.3)	110433 (28.2)	117462 (29.6)	120801 (30.6)	124391 (31.9)	<0.001**
**Alcohol consumption, n (%)**													
No alcohol consumption	82147 (70.0)	93138 (70.9)	108256 (70.7)	129886 (70.5)	137741 (71.0)	151994 (71.6)	169619 (69.5)	159519 (65.5)	160084 (64.4)	166452 (64.2)	161344 (63.7)	167135 (63.9)	<0.001**
Low risk alcohol consumption	31721 (27.0)	34600 (26.3)	41045 (26.8)	50310 (27.3)	52133 (26.9)	56258 (26.5)	70112 (28.7)	79852 (32.8)	84434 (34.0)	88968 (34.3)	88419 (34.9)	91182 (34.8)	<0.001**
High risk alcohol consumption	3492 (2.98)	3578 (2.72)	3781 (2.47)	4069 (2.21)	4021 (2.07)	4038 (1.90)	4328 (1.77)	4303 (1.77)	3977 (1.60)	3803 (1.47)	3525 (1.39)	3335 (1.27)	<0.001**
**Comorbidities, n (%)**													
Hypertension	222302 (74.1)	237453 (74.7)	253054 (75.4)	269075 (75.8)	281742 (76.2)	294236 (76.5)	304925 (77.1)	311876 (77.5)	312206 (77.8)	315212 (78.0)	312336 (78.0)	308599 (78.3)	<0.001**
Hypercholesterolemia	169551 (56.5)	185959 (58.5)	205119 (61.1)	224327 (63.2)	239694 (64.9)	253181 (65.8)	266431 (67.4)	274067 (68.1)	275969 (68.8)	278764 (69.0)	277187 (69.3)	274519 (69.6)	<0.001**
Obesity	75824 (45.2)	77715 (45.1)	81900 (45.4)	89403 (45.6)	91969 (45.5)	97538 (45.4)	112956 (46.3)	119249 (46.4)	121540 (46.1)	125400 (46.0)	123006 (45.3)	122075 (44.8)	<0.001**
Retinopathy	14466 (4.8)	16976 (5.3)	19892 (5.9)	22930 (6.5)	25440 (6.9)	28177 (7.3)	31020 (7.8)	34166 (8.5)	36446 (9.1)	38403 (9.5%)	39175 (9.79)	40676 (10.3)	<0.001**
Chronic kidney Disease	60473 (20.2)	68120 (21.4)	77217 (23.0)	84019 (23.7)	87471 (23.7)	96813 (25.2)	103750 (26.2)	111530 (27.7)	116389 (29.0)	117985 (29.2)	121388 (30.3)	124078 (31.5)	<0.001**
Cardiovascular disease	55035 (18.4)	61252 (19.3)	67826 (20.2)	74674 (21.0)	80154 (21.7)	85780 (22.3)	90173 (22.8)	93578 (23.3)	94779 (23.6)	97136 (24.0)	97202 (24.3)	96379 (24.4)	<0.001**
Heart failure	13498 (4.5)	15275 (4.8)	17176 (5.1)	19653 (5.5)	21852 (5.9)	24623 (6.4)	27012 (6.8)	28664 (7.1)	29331 (7.3)	30218 (7.5)	29777 (7.4)	28870 (7.3)	<0.001**
**Clinical variables, mean, (SD)**													
Systolic blood pressure (mm Hg)	138 (16.9)	137 (16.5)	136 (16.1)	135 (15.6)	135 (15.3)	134 (14.8)	133 (14.4)	133 (14.2)	133 (13.9)	133 (13.6)	133 (13.8)	133 (13.7)	<0.001*
Diastolic blood pressure (mm Hg)	76.4 (9.79)	76.1 (9.78)	75.8 (9.81)	75.5 (9.82)	75.2 (9.84)	74.7 (9.74)	74.4 (9.77)	74.5 (9.77)	74.7 (9.73)	74.9 (9.68)	75.0 (9.77)	75.1 (9.74)	<0.001*
BMI >30 kg/m^2^	75824 (45.2)	77715 (45.1)	81900 (45.4)	89403 (45.6)	91969 (45.5)	97538 (45.4)	112956 (46.3)	119249 (46.4)	121540 (46.1)	125400 (46.0)	123006 (45.3)	122075 (44.8)	<0.001*
BMI, (kg/m^2^)	30.1 (5.02)	30.1 (5.01)	30.1 (5.05)	30.1 (5.07)	30.1 (5.08)	30.1 (5.11)	30.2 (5.18)	30.2 (5.19)	30.2 (5.20)	30.2 (5.22)	30.1 (5.21)	30.0 (5.21)	<0.001*
HbA1c, (%)	7.16 (1.46)	7.23 (1.48)	7.25 (1.47)	7.17 (1.36)	7.25 (1.36)	7.22 (1.33)	7.09 (1.31)	7.07 (1.29)	7.10 (1.29)	7.10 (1.31)	7.07 (1.29)	7.09 (1.29)	<0.001*
Cholesterol Total,(mg/dL)	194 (39.5)	192 (39.9)	193 (39.9)	190 (39.8)	188 (39.3)	187 (39.4)	184 (39.4)	184 (39.2)	183 (39.7)	182 (39.8)	183 (40.5)	182 (40.4)	<0.001*
Cholesterol HDL,(mg/dL)	50.0 (13.3)	49.9 (13.3)	49.1 (13.2)	48.6 (12.9)	48.9 (13.2)	49.0 (13.2)	49.4 (13.2)	49.0 (13.1)	48.9 (13.0)	48.9 (13.0)	49.3 (13.1)	48.7 (12.7)	<0.001*
Cholesterol LDL,(mg/dL)	115 (33.0)	113 (33.3)	114 (33.3)	112 (33.0)	109 (32.8)	109 (32.8)	105 (32.7)	105 (32.5)	105 (32.7)	103 (32.5)	103 (33.1)	103 (33.3)	<0.001*
Triglycerides, (mg/dL)	153 (110)	155 (108)	157 (107)	153 (103)	155 (104)	154 (103)	156 (102)	157 (103)	158 (103)	159 (105)	162 (106)	159 (104)	<0.001*
Estimated glomerular filtration rate (mL/min/1.73m^2^)	73.9 (17.0)	73.5 (17.3)	73.0 (17.6)	73.6 (17.5)	74.3 (17.6)	73.8 (17.8)	73.4 (17.9)	72.8 (18.2)	72.7 (18.4)	73.7 (18.4)	73.5 (18.5)	73.4 (18.5)	<0.001*
Albumin/creatinine rat (mg/g)	38.0 (138)	39.3 (141)	39.7 (149)	41.6 (158)	40.7 (153)	42.6 (158)	44.1 (165)	48.7 (181)	54.3 (202)	58.2 (211)	62.3 (223)	64.4 (227)	<0.001*
**Drug treatments, n (%)**													
Antidiabetic drugs	206701 (68.9)	222751 (70.0)	238624 (71.1)	257162 (72.4)	271761 (73.5)	284746 (74.0)	292978 (74.1)	300237 (74.6)	305678 (76.2)	312368 (77.3)	316072 (79.0)	319272 (81.0)	<0.001**
Antithrombotic drugs	113108 (37.7)	121593 (38.3)	132746 (68.2)	140210 (39.5)	145230 (39.3)	141294 (36.7)	148350 (37.5)	148959 (37.0)	147561 (36.8)	147479 (36.5)	144272 (36.1)	139478 (35.4)	<0.001**
Lipid-lowering drugs	124280 (41.4)	136850 (43.0)	155808 (46.4)	173200 (48.8)	186254 (50.4)	189858 (49.3)	203642 (51.5)	207653 (51.6)	208937 (52.1)	209943 (51.9)	208987 (52.2)	208840 (53.0)	<0.001**
Antihypertensive	189348 (63.1)	200707 (63.1)	215455 (64.2)	229767 (64.7)	240860 (65.2)	247106 (64.2)	261151 (66.0)	268779 (66.8)	271849 (67.8)	276935 (68.5)	278346 (69.6)	278356 (70.6)	<0.001**

BMI, body mass index; HbA1c, glycated hemoglobin A1c; SD, standard deviation; *Anova 1 Factor; **Chi-square trend test.

Concerning comorbidities, hypertension and dyslipidemia were highly prevalent among T2DM subjects; both increased over the years. The prevalence of chronic diabetic complications also increased progressively. We observed a progressive decrease in BP and LDL-C mean values while BMI and HbA1c did not show any relevant changes.

### Use of Antidiabetic Treatment

**Figure 1:**
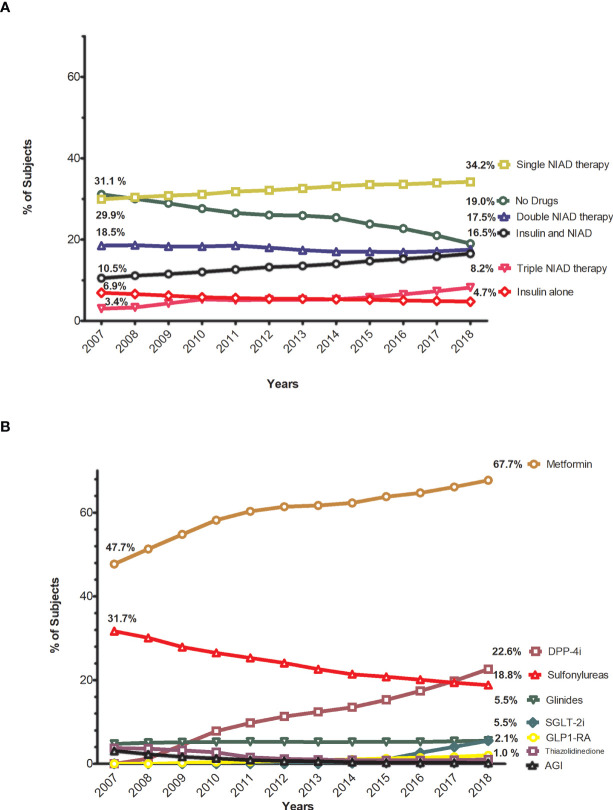
Trends in the proportion of subjects by different steps of antidiabetic treatment and using non-insulin antidiabetic drugs. **(A)** Annual trends in the proportion of subjects by steps of antidiabetic treatment. **(B)** Annual trends in the proportion of subjects treated with non-insulin antidiabetice drugs.

**Figure 1A** and **Supplementary Table 1** show the proportion of patients in each treatment step (no drugs, NIAD monotherapy, NIAD dual therapy, NIAD triple therapy, insulin alone, and insulin in combination). We observed a considerable decrease (12.1%) in people without pharmacological antidiabetic treatment, from 29.9% to 19.0% at the end of the observation period. NIAD monotherapy increased from 29.9% to 34.2%. The use of double NIAD therapy and insulin alone slightly decreased, while triple NIAD therapy and insulin in combination with NIAD increased over the years.

**Figure 1B** and **Supplementary Table 1** show the frequencies of antidiabetic drug use. The use of metformin increased from 47.7% to 67.7%, as was the case for DPP-4i: from 0 to 22.6%. The use of sodium-glucose co-transporter 2 inhibitors (SGLT-2i) and GLP-1ra increased over the years, but their use remained low (5.5% and 2.1% of patients, respectively, in 2018). The use of thiazolidinediones, alpha-glucosidase inhibitors, and especially sulphonylureas decreased notably (the latter from 31.7% to 18.8%), while the use of glinides remained stable (5.5% in 2018).

**Figure 2:**
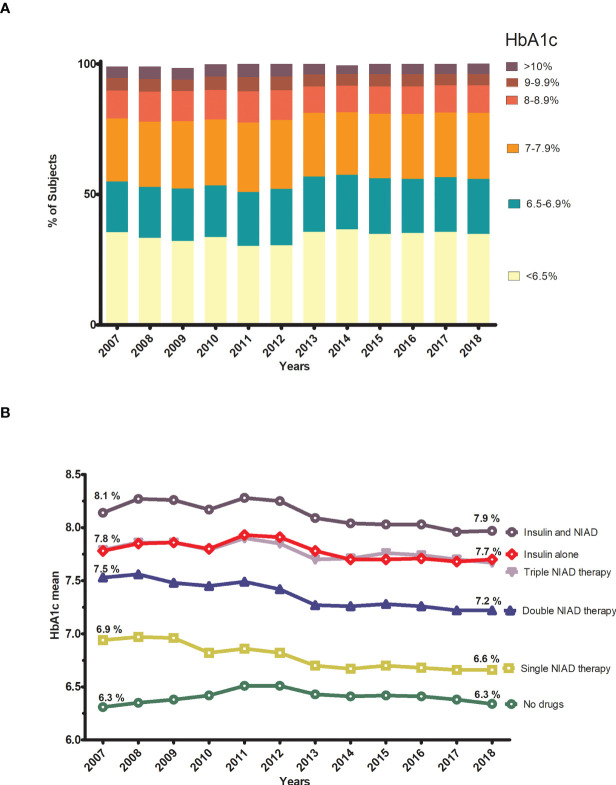
Trends in glycemic control by HbA1c categories and by step of treatment. **(A) **Annual trends in distribution of subjects according to HbA1c categories. **(B)** Annual trends in the mean HbA1c for each step of treatmet.

Trends in HbA1c levels are presented in **Figure 2** and **Supplementary Tables 2**, **3**. Small changes were observed throughout the study period for the different categories of HbA1c: from 54.9% to 55.9% for the HbA1c<7% threshold and from 79% to 81.1% for HbA1c<8% (**Figure 2A**). At the end of the observational period, more than a third of subjects (34.9%) had an HbA1c<6.5% (47.5 mmol/mol) while 18.9% were poorly controlled (HbA1c>8%, 64 mmol/mol). When we analyzed the mean HbA1c for each step of treatment (i.e., no drugs, NIAD monotherapy, NIAD dual therapy, NIAD triple therapy, insulin alone and insulin in combination), in general, glycemic control worsened with the more complex treatments, but a trend to improvement was observed for all drug strategies from 2013 to 2018 (**Figure 2B**). The more pronounced reductions of mean HbA1c were observed in those subjects receiving double NIAD who experienced a reduction of mean HbA1c of -0.33% (from 7.5% to 7.2%) and in those under monotherapy treatment with a reduction of -0.28% (from 6.9% to 6.6%), while non-pharmacological treatment (no drugs) remained stable (6.3%).

### Use of Antithrombotic, Antihypertensive, and Lipid-Lowering Agents

**Figure 3:**
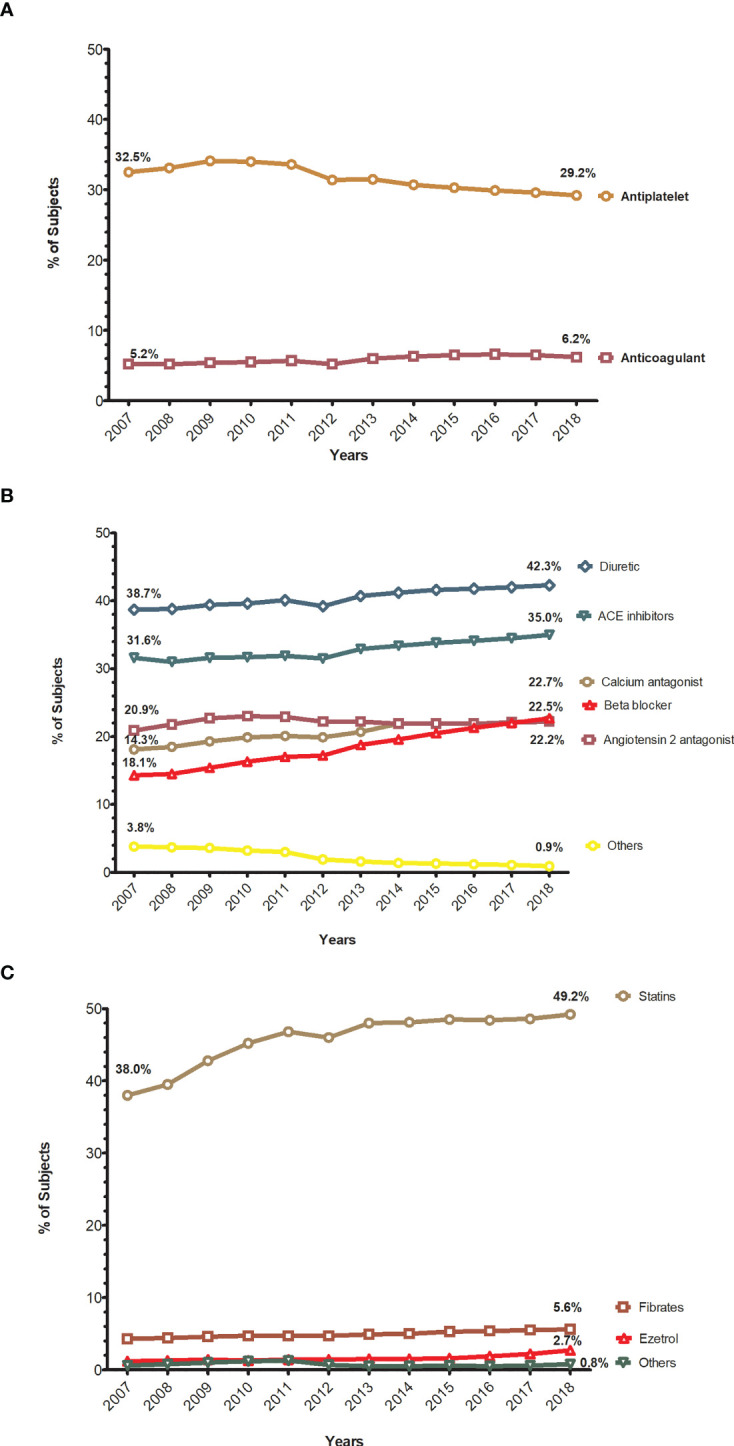
Trends in the proportion of subjects treated with antithrombotic, antihypertensive, and lipid-lowering drugs. **(A)** Annual trends in the proportion of subjects treated with antithrombotic drugs. **(B)** Annual trends in the proportion of subjects treated with antihypertensive drugs. **(C)** Annual trends in the proportion of subjects treated with lipid lowering drugs.

**Figure 3** and **Supplementary Table 4** show the use of antithrombotic, antihypertensive, and lipid-lowering drugs over the years. During the study period, the use of antihypertensive increased from 63.1% in 2007 to 70.6% in 2018, the use of lipid-lowering drugs increased from 41.4% to 53.0%, while the use of antithrombotic drugs slightly decreased (from 37.7% to 35.4%) (**Table 1**). The use of antiplatelet drugs slightly decreased over the years (from 32.5% to 29.2%), while the use of anticoagulants increased (5.2% to 6.2%) (**Figure 3A**). Among the antihypertensive drugs, renin-angiotensin system (RAS) drugs (Angiotensin-converting-enzyme inhibitors-ACEI or Angiotensin II receptor blocker-ARB) and diuretics were principally used and gradually increased (from 52.5% to 57.2% and from 38.7% to 42.3%, respectively) (**Figure 3B**). There was an increase in the use of ACEI, ARB, and especially beta-blockers (1.3%, 3.4%, and 8.4%, respectively). Regarding the lipid-lowering agents, statins were by far the most widely used lipid-lowering drugs in all years, and their use greatly increased (11.2%), from 38.0% to 49.2%, at the end of the study period (**Figure 3C**).

### Therapeutic Goals

**Figure 4:**
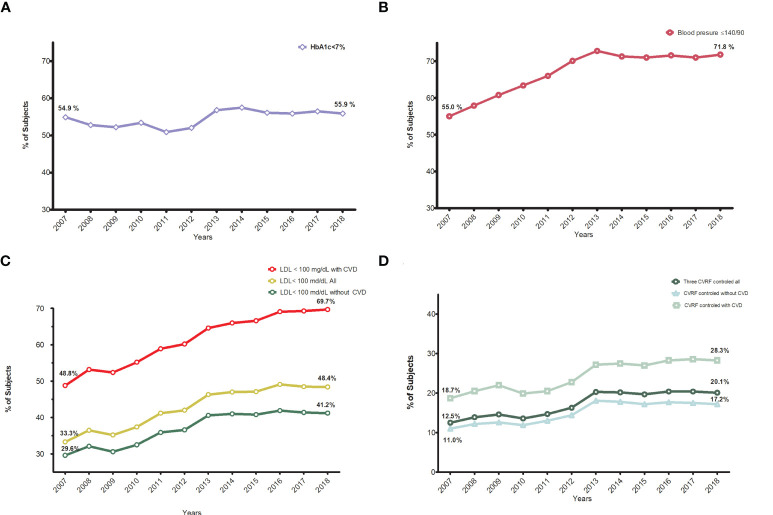
Trends in the proportion of subjects achieving different therapeutic goals. **(A)** Annual trends in the proportion of subjects reaching HbA1c <7%. **(B)** Annual trends in the proportion of subjects reaching blood pressure ≤140/90 mmHg. **(C)** Annual trends in the proportion of subjects reaching LDL-C<100 mg/dl, global,with and without cardiovascular disease. **(D)** Annual trends in the proportion of subjects reaching all three goals, global, with and without cardiocvascular disease.

Trends in achieving the therapeutic goals are presented in **Figure 4** and **Supplementary Tables 5**, **6**. The percentage of subjects achieving an HbA1c target <7% (53.0 mmol/mol) remained fairly stable over the years, with some smooth oscillations: the percentage of subjects meeting this goal was lowest in 2011 (50.9%) and highest in 2014 (57.5%), and at the end of the observation period it was 55.9% (**Figure 4A**). According to the threshold of our institution (HbA1c<8%), there was a 2% increase, from 79.0% to 81.1%. These results are presented in the **Supplementary Table 5**.

Major improvements were seen in the proportion of people with blood pressure control (≤140/90 mm Hg), from 55% to 71.8% (**Figure 4B**). The same trend was seen with lipid control (low-density lipoprotein cholesterol <100 mg/dl): from 33.4% to 48.4%, especially in people with CVD: 48.8 to 69.7% (**Figure 4C**). Finally, achievement of all three targets (combined indicator) improved from 12.5% to 20.1% in the whole population and from 18.7% to 28.3% in those with CVD (**Figure 4D**).

Improvements in all indicators, except LDL-C in patients with CVD, plateaued after 2013. The percentage of patients with CVD with LDL-C control continued increasing from 64.6% in 2013 to 69.7% in 2018.

### Trend Tests Analysis

We observed significant p values for trend test for nearly all of the variables over the years, except for the distribution of subjects according to HbA1c categories and the use of angiotensin receptor blockers.

## Discussion

This cross-sectional study shows the trends in the degree of cardiovascular risk factor control and prescribing practices between 2007 and 2018 in primary care in a Mediterranean area. During this period, a 31% increase (94,411 subjects) was observed in the registered T2DM population, and in the prevalence of cardiovascular disease, heart failure, and chronic kidney disease (from 18.4 to 24.4%, from 4.5 to 7.3% and from 20.2 to 31.3%, respectively). This could be probably due to an improvement in registration and the aging population. In fact, there was an increase in the mean age (from 68.4 to 70.3 years) and in the percentage of patients older than 75 (from 32.7% to 37.6%). The unexpected low diabetes duration could be explained by the EMR system (eCAP), which was widely introduced in 2006 in Catalonia. The accuracy of patients’ diagnosis dates reported in the first years could be affected by memory, while further diagnoses based on laboratory results were probably more accurately registered after the implementation of the eCAP. There was also an increase in the records of chronic complications, especially chronic kidney disease (CKD), retinopathy, and heart failure. At the end of the study, nearly a quarter of patients (24.4%) had CVD and 31% CKD. These figures align with those of other studies on the prevalence of chronic complications (21, 22).

Concerning the control of CVRFs, greater improvements have been observed for cholesterol and BP than for glycemic control, as described in previous reports in the US and the UK (8–12). In our database, glycemic control slightly improved from 54.9% to 55.9% for the HbA1c<7% threshold and from 79 to 81.1% for HbA1c<8%, which is the pay-for-performance goal of our institution (ICS). These changes do not seem to be outstanding, but at least they have not decreased as recently described in the US (10), in Germany and Austria (15), and in an international collaboration study of 49 countries (16). Moreover, regarding the treatment steps, a slight trend in the reduction in the mean HbA1c was seen in all pharmacological steps, the greatest seen with one or two NIAD starting in 2013, with a 0.3% reduction in both cases.

Major improvements were seen in the percentage of people achieving target blood pressure, from 55% to 71.8%. The same trend was seen for LDL-C targets: 33.4% to 48.4%, especially in people with CVD: 48.8% to 69.7%. Nevertheless, only one in five patients achieved all three risk factor control goals, increasing from 12.5% to 20.1% in the whole population, but stagnation occurred after 2013. This improvement was greater for those with CVD (from 18.7% to 28.3%), probably due to more intense treatment with lipid-lowering drugs. Published data from the US, based on NHANES surveys from 1999 to 2018, showed a worsening of glycemic control (HbA1c<7%) between the 2007–2010 and 2015–2018 surveys: from 57.4% to 50.5% of people achieving this target (10). In the same study, the percentage of people with blood pressure control (BP <140/90 mmHg) decreased from 74.2% to 70.4%, and minimal improvements were seen in lipid control, while the percentage of people in whom all three targets were simultaneously achieved plateaued after 2010 at around 22.2% in 2015–2018 (10). This percentage in the composite indicator is similar to the 20% observed in our database that also plateaued from 2013 to 2018.

A matter of concern is the fact that the composite indicator plateaued after an initial and progressive improvement. A possible explanation would be a ceiling on glycemic and blood pressure improvements, beyond which it is difficult to improve since relevant factors such as patient adherence, tolerance to drugs, and especially the need for less stringent goals in patients of advanced age. More than a third of the subjects were 75 years or older, and the benefits of strict glycemic control this population has not yet been fully demonstrated (23, 24). Overcoming clinical inertia by healthcare professionals, improving adherence to medications and healthy lifestyle behaviors in patients, and providing necessary health care access and resources, education, and self-management support by the healthcare system are challenges that need to be tackled (25).

Regarding pharmacological treatment, there was an increase in the proportion of subjects receiving antidiabetic drugs, especially for metformin and DPP4i. The use of newer classes of glucose-lowering drugs rose, whereas older classes such as sulfonylureas, pioglitazone, and alfa glucosidase inhibitors declined; these findings reflect a shift toward safer or better-tolerated drugs. Although increasing, the use of SGLT-2i and GLP-1ra agents with demonstrated cardiovascular benefits (2–7), remained low, probably because they are newer, more expensive, and have prescription restrictions in our country. For instance, there were negative economic incentives during this period for the prescription of SGLT-2i and GLP-1ra that may have contributed to their limited use. In addition, in Spain, GLP-1ra are only reimbursed, after administrative validation, in combination with other antidiabetic drugs for subjects with a BMI>30. This was not the case for SGLT-2i despite a more recent introduction in our country (from 2013 on). The increase after 2015 was very impressive, quickly surpassing the GLP-1ra prescription rate in 2018 (5.5% vs. 2.1%, respectively). DPP4i had the greatest increase in use, in agreement with other reports conducted worldwide (8–10, 13–18). They are an alternative to sulfonylureas for their lower risk of hypoglycemia, bodyweight increase, and greater convenience as an oral treatment instead of injectable drugs (26).

The percentage of people treated with statins, beta-blockers, and RAS drugs increased notably. Similar figures have been observed in the NHANES (National Health and Nutrition Examination Survey) study as mentioned earlier: 56.3% used statins, and 60.3% used a RAS antihypertensive (10). Use of antithrombotic therapies (mainly aspirin) showed a slight reduction, probably because of the recommendation against the prescription of aspirin in primary prevention since 2010 (27).

This study has some strengths and limitations. The main strength is that we used a large database to determine trends in primary care. However, this was a retrospective study, and missing data should be noted, especially in the first years of the study. For instance, the percentage of missing values for HbA1c was 36.9% in 2007, which decreased to 25% in 2018. This was also the case for the available values for calculating the combined 3 CVRF indicators: only 48.9% of patients had both three measurements in 2007 but this increased to 62.1% in 2018. Lack of data could be explained due to some patients not attending their routine visits, incomplete recording of patient information by some health professionals, and that a small proportion of subjects are under the care of endocrinologists in hospitals or private clinics. In studies like the current one that include a very large sample of participants, even small differences are statistically significant. Actually, the statistical differences found in our study do not add relevant information for the final interpretation of the findings. Nevertheless, a large number of measurements and the consistency of similar annual results contribute to the validity of our conclusions.

In conclusion, in our country, during 2007-2018, relevant improvements were observed in blood pressure and lipid control, especially in people with cardiovascular disease. Despite the increase in the use of antidiabetic and cardiovascular drugs, the proportion of patients in which the three objectives were simultaneously achieved is still insufficient and plateaued after 2013. Finally, the use of antidiabetic drugs with demonstrated cardio renal benefits like SGLT-2 and GLP-1ra increased over the years, but their use remained very low.

## Data Availability Statement

The data analyzed in this study is subject to the following licenses/restrictions: The data controller for SIDIAP does not allow the sharing of raw data. Requests to access these datasets should be directed to JF-N, josep.franch@gmail.com.

## Ethics Statement

The studies involving human participants were reviewed and approved by Institutional Review Board (or Ethics Committee) of IDIAP Jordi Gol i Gurina Foundation (protocol code 21/111-P and date of approval 04/05/2021). Written informed consent for participation was not required for this study in accordance with the national legislation and the institutional requirements.

## Author Contributions

MM-C and BV have contributed equally to this work and share the first authorship. MM-C, JF-N, JR, RP-T, MB, DM, and BV conceived the research and participated in its design. JR and RP-T performed statistical analysis. MM-C and BV wrote the initial draft of the manuscript, which MM-C, JF-N, JR, RP-T, MB, DM, and BV edited. All authors contributed to the article and approved the submitted version.

## Conflict of Interest

MM-C has received advisory honorarium from Astra-Zeneca, Bayer, Boehringer Ingelheim, GSK, Lilly, MSD, Novartis, Novo Nordisk, and Sanofi; he has received speaker honorarium from Astra-Zeneca, Bayer, Boehringer Ingelheim, GSK, Lilly, Menarini, MSD, Novartis, Novo Nordisk, and Sanofi; he has received research grants to the institution from Astra-Zeneca, GSK, Lilly, MSD, Novartis, Novo Nordisk and Sanofi. JF-N has received advisory and or speaking fees from Astra-Zeneca, Ascensia, Boehringer Ingelheim, GSK, Lilly, MSD, Novartis, Novo Nordisk, and Sanofi; he has received research grants to the institution from Astra-Zeneca. GSK, Lilly, MSD, Novartis, Novo Nordisk, Sanofi, and Boehringer. DM has received advisory and/or speaking fees from Astra-Zeneca, Ascensia, Boehringer Ingelheim, GSK, Lilly, MSD, Novartis, Novo Nordisk, and Sanofi; he has received research grants to the institution from Astra-Zeneca, GSK, Lilly, MSD, Novartis, Novo Nordisk, Sanofi, and Boehringer. MB has received advisory and speaking fees from MSD.

The remaining authors declare that the research was conducted in the absence of any commercial or financial relationships that could be construed as a potential conflict of interest.

## Publisher’s Note

All claims expressed in this article are solely those of the authors and do not necessarily represent those of their affiliated organizations, or those of the publisher, the editors and the reviewers. Any product that may be evaluated in this article, or claim that may be made by its manufacturer, is not guaranteed or endorsed by the publisher.
