# Supplementary material for: Trends in the Degree of Control and Treatment of Cardiovascular Risk Factors in People With Type 2 Diabetes in a Primary Care Setting in Catalonia During 2007–2018
Source: Front Endocrinol (Lausanne). 2022 Jan 10;12:810757. doi: 10.3389/fendo.2021.810757 (PMC8784522; doi:10.3389/fendo.2021.810757)
Supplement: Supplementary file 1 [file DataSheet_1.docx]

**Supplementary material**

**Trends in the degree of control and treatment of cardiovascular risk factors in people with type 2 diabetes in the primary care setting in Catalonia during 2007-2018**

Manel Mata-Cases MD, PhD, Bogdan Vlacho PharmD, MSc, Jordi Real PhD, Ramon Puig-Treserra, Magdalena Bundó MD, Josep Franch-Nadal MD, PhD, Dídac Mauricio MD, PhD

| **Table of Contents** | | **Page** |
| --- | --- | --- |
| **Supplementary Table 1** | Trends in the steps of antidiabetic treatment and prescribed antidiabetic drug classes | 2 |
| **Supplementary Table 2** | Distribution of subjects according to HbA1c categories | 3 |
| **Supplementary Table 3** | Trends in mean HbA1c by different steps of treatment | 4 |
| **Supplementary Table 4** | Trends in the use of cardiovascular drugs | 5 |
| **Supplementary Table 5** | Trends in the control of blood bressure, HbA1c, and LDL-cholesterol in patients with and without cardiovascular disease | 6 |
| **Supplementary Table 6** | Trends in the composite indicator of the three cardiovascular risk factors (blood pressure, HbA1c and LDL-cholesterol) in patients with and without cardiovascular disease | 7 |

Supplementary table 1. Trends in the steps of antidiabetic treatment and prescribed antidiabetic drug classes

|  | **2007** | **2008** | **2009** | **2010** | **2011** | **2012** | **2013** | **2014** | **2015** | **2016** | **2017** | **2018** | **p-value*** |
| --- | --- | --- | --- | --- | --- | --- | --- | --- | --- | --- | --- | --- | --- |
| **N** | **299855** | **318065** | **335771** | **355019** | **369600** | **384826** | **395470** | **402312** | **401175** | **404252** | **400209** | **394266** | **-** |
| **Steps of antidiabetic treatment** |  |  |  |  |  |  |  |  |  |  |  |  |  |
| No antidiabetic drugs, n (%) | 93154 (31.1) | 95314 (30.0) | 97147 (28.9) | 97857 (27.6) | 97839 (26.5) | 100080 (26.0) | 102492 (25.9) | 102075 (25.4) | 95497 (23.8) | 91884 (22.7) | 84137 (21.0) | 74994 (19.0) | <0.001 |
| NIAD monotherapy, n (%) | 89703 (29.9) | 96700 (30.4) | 103390 (30.8) | 110543 (31.1) | 117488 (31.8) | 123716 (32.1) | 129026 (32.6) | 132978 (33.1) | 134536 (33.5) | 135905 (33.6) | 135623 (33.9) | 134787 (34.2) | <0.001 |
| NIAD double therapy, n (%) | 55608 (18.5) | 59220 (18.6) | 61502 (18.3) | 64873 (18.3) | 68239 (18.5) | 69461 (18.0) | 68816 (17.4) | 68430 (17.0) | 68008 (17.0) | 68202 (16.9) | 68333 (17.1) | 68819 (17.5) | <0.001 |
| NIAD triple therapy, n (%) | 9101  (3.04) | 10459 (3.29) | 14506 (4.32) | 18650 (5.25) | 18772 (5.08) | 20127 (5.23) | 20525 (5.19) | 21239 (5.28) | 23298 (5.8) | 26269 (6.5) | 29118 (7.3) | 32143 (8.2) | <0.001 |
| Insulin monotherapy, n (%) | 20872  (6.9) | 20954  (6.6) | 20645  (6.2) | 20426  (5.8) | 20601  (5.6) | 20745  (5.4) | 21149  (5.4) | 21203  (5.3) | 20847  (5.2) | 20386  (5.0) | 19699  (4.9) | 18636  (4.7) | <0.001 |
| Insulin with NIAD combination, n (%) | 31417 (10.5) | 35418 (11.1) | 38581 (11.5) | 42670 (12.0) | 46661 (12.6) | 50697 (13.2) | 53462 (13.5) | 56387 (14.0) | 58989 (14.7) | 61606 (15.2) | 63299 (15.8) | 64887 (16.5) | <0.001 |
| **Non-insulin antidiabetic drugs** |  |  |  |  |  |  |  |  |  |  |  |  |  |
| Metformin, n(%) | 142917 (47.7) | 163130 (51.3) | 183959 (54.8) | 206545 (58.2) | 222695 (60.3) | 236151 (61.4) | 243810 (61.7) | 250611 (62.3) | 255884 (63.8) | 261498 (64.7) | 264391 (66.1) | 266881 (67.7) | <0.001 |
| Sulphonylureas, n (%) | 95178 (31.7) | 95759 (30.1) | 93704 (27.9) | 93982 (26.5) | 93359 (25.3) | 92908 (24.1) | 89372 (22.6) | 86028 (21.4) | 83633 (20.8) | 81238 (20.1) | 77704 (19.4) | 74217 (18.8) | <0.001 |
| Glinides, n (%) | 14393  (4.8) | 16018  (5.0) | 17343  (5.2) | 18566  (5.2) | 19535  (5.3) | 20321  (5.3) | 20622  (5.2) | 20996  (5.2) | 20990  (5.2) | 21263  (5.2) | 21608  (5.4) | 21832  (5.5) | <0.001 |
| Thiazolidinediones, n (%) | 11333  (3.8) | 11558  (3.6) | 10829  (3.2) | 9768  (2.8) | 5758  (1.6) | 4577  (1.2) | 3920  (0.9) | 3610  (0.9) | 3454  (0.9) | 3465  (0.9) | 3709  (0.9) | 3972  (1.0) | <0.001 |
| SGLT-2i, n (%) | - | - | - | - | - | - | - | 649  (0.16) | 4270  (1.0) | 10706  (2.7) | 16291  (4.1) | 21830  (5.5) | <0.001 |
| DPP-4i, n (%) | - | 4013  (1.3) | 15064  (4.5) | 27734  (7.8) | 35983  (9.7) | 43476 (11.3) | 48882 (12.4) | 54359 (13.5) | 61412 (15.3) | 70226 (17.4) | 79369 (19.8) | 88927 (22.6) | <0.001 |
| GLP-1ra, n (%) | - | 29  (0.01) | 795  (0.2) | 1515  (0.4) | 1875  (0.5) | 2799  (0.7) | 3428  (0.9) | 4316  (1.1) | 5163  (1.3) | 6079  (1.5) | 6831  (1.7) | 8189  (2.1) | <0.001 |
| AGI, n (%) | 9147  (3.1) | 7418  (2.3) | 5767  (1.7) | 4495  (1.3) | 3476  (0.9) | 2776  (0.7) | 2180  (0.6) | 1707  (0.4) | 1375  (0.3) | 1098  (0.3) | 909  (0.3) | 673  (0.2) | <0.001 |

* Chi-square trend test

Supplementary table 2. Distribution of subjects according to HbA1c categories

|  | **2007** | **2008** | **2009** | **2010** | **2011** | **2012** | **2013** | **2014** | **2015** | **2016** | **2017** | **2018** | **p-value*** |
| --- | --- | --- | --- | --- | --- | --- | --- | --- | --- | --- | --- | --- | --- |
| Patients with available HbA1c, n (%) | 189078  (63.1) | 203420  (64.0) | 219979  (65.5) | 230458  (64.9) | 245642  (66.5) | 267872  (69.6) | 279531  (70.7) | 287588  (71.5) | 294391  (73.4) | 300135  (74.2) | 301135  (75.2) | 303524  (77.0) | - |
| HbA1c <6.5%, n (%) | 67123 (35.5) | 67739 (33.3) | 70613 (32.1) | 77639 (33.7) | 74259 (30.2) | 81448 (30.4) | 99515 (35.6) | 105203 (36.6) | 102826 (34.9) | 105685 (35.2) | 107158 (35.6) | 105845 (34.9) | 0.0579 |
| HbA1c 6.5-6.9%, n (%) | 36681 (19.4) | 39 667 (19.5) | 44216 (20.1) | 45459 (19.7) | 50762 (20.7) | 57953 (21.6) | 59174 (21.2) | 59757 (20.8) | 62385 (21.2) | 62069 (20.7) | 62977 (20.9) | 63663 (21.0) | 0.0579 |
| HbA1c 7-7.9%, n (%) | 45568 (24.1) | 50855 (25.0) | 56755 (25.8) | 58252 (25.3) | 65442 (26.6) | 71034 (26.5) | 68183 (24.4) | 69313 (24.1) | 73054 (24.8) | 74713 (24.9) | 74662 (24.8) | 76740 (25.3) | 0.0579 |
| HbA1c 8-8.9%, n (%) | 20420 (10.8) | 23393 (11.5) | 25341  (11.5) | 26120 (11.3) | 29177 (11.9) | 30546 (11.4) | 28245 (10.1) | 28928 (10.1) | 30638 (10.4) | 31559 (10.5) | 31171 (10.4) | 31784 (10.5) | 0.0579 |
| HbA1c 9-9.9%, n (%) | 10210 (5.4) | 10985 (5.4) | 11087 (5.0) | 12061 (5.23) | 13664 (5.56) | 14150 (5.28) | 12905 (4.62) | 12900 (4.49) | 13654 (4.64) | 13876 (4.62) | 13642 (4.53) | 13767 (4.54) | 0.0579 |
| HbA1c >10%, n (%) | 9076  (4.8) | 10781 (5.3) | 11967 (5.4) | 10927 (4.74) | 12338 (5.02) | 12741 (4.76) | 11509 (4.12) | 11487 (3.99) | 11834 (4.02) | 12233 (4.08) | 11525 (3.83) | 11725 (3.86) | 0.0579 |

HbA1c: glycated hemoglobin A1c; * Chi-square trend test

Supplementary table 3. Trends in mean HbA1c by different steps of treatment

|  | **2007** | **2008** | **2009** | **2010** | **2011** | **2012** | **2013** | **2014** | **2015** | **2016** | **2017** | **2018** | **p-value*** |
| --- | --- | --- | --- | --- | --- | --- | --- | --- | --- | --- | --- | --- | --- |
| Patients with HbA1c available, n (%) | 189078  (63.1) | 203420  (64.0) | 219979  (65.5) | 230458  (64.9) | 245642  (66.5) | 267872  (69.6) | 279531  (70.7) | 287588  (71.5) | 294391  (73.4) | 300135  (74.2) | 301135  (75.2) | 303524  (77.0) | - |
| Non- drugs**,** HbA1c %, mean, (SD) | 6.31 (0.99) | 6.35 (0.96) | 6.38 (0.96) | 6.42 (0.94) | 6.51 (0.92) | 6.51 (0.89) | 6.43 (0.90) | 6.41 (0.89) | 6.42 (0.87) | 6.41 (0.89) | 6.38 (0.87) | 6.34 (0.83) | <0.001 |
| Single NIAD, HbA1c %, mean, (SD) | 6.94 (1.28) | 6.97 (1.26) | 6.96 (1.24) | 6.82 (1.07) | 6.86 (1.02) | 6.82 (0.98) | 6.70 (0.96) | 6.67 (0.94) | 6.70 (0.94) | 6.68 (0.94) | 6.66 (0.92) | 6.66 (0.91) | <0.001 |
| Double NIAD, HbA1c %, mean, (SD) | 7.53 (1.47) | 7.56 (1.47) | 7.48 (1.46) | 7.45 (1.33) | 7.49 (1.32) | 7.42 (1.29) | 7.27 (1.25) | 7.26 (1.25) | 7.28 (1.25) | 7.26 (1.26) | 7.22 (1.23) | 7.22 (1.24) | <0.001 |
| Triple NIAD, HbA1c %, mean, (SD) | 7.79 (1.61) | 7.86 (1.62) | 7.86 (1.66) | 7.79 (1.38) | 7.90 (1.36) | 7.85 (1.36) | 7.70 (1.33) | 7.71 (1.33) | 7.76 (1.33) | 7.74 (1.33) | 7.70 (1.31) | 7.67 (1.29) | <0.001 |
| Insulin alone, HbA1c %, mean, (SD) | 7.78 (1.60) | 7.85 (1.62) | 7.86 (1.66) | 7.80 (1.52) | 7.93 (1.55) | 7.91 (1.53) | 7.78 (1.53) | 7.70 (1.50) | 7.70 (1.47) | 7.71 (1.50) | 7.68 (1.46) | 7.70 (1.49) | <0.001 |
| Insulin and NIAD, HbA1c %, mean, (SD) | 8.14 (1.59) | 8.27 (1.63) | 8.26 (1.64) | 8.17 (1.55) | 8.28 (1.56) | 8.25 (1.55) | 8.09 (1.54) | 8.04 (1.51) | 8.03 (1.52) | 8.03 (1.53) | 7.96 (1.50) | 7.97 (1.50) | <0.001 |

HbA1c: glycated hemoglobin A1c; NIAD: non-insulin antidiabetic drug; * Anova 1 Factor

Supplementary table 4. Trends in the use of cardiovascular drugs

|  | **2007** | **2008** | **2009** | **2010** | **2011** | **2012** | **2013** | **2014** | **2015** | **2016** | **2017** | **2018** | **p-value*** |
| --- | --- | --- | --- | --- | --- | --- | --- | --- | --- | --- | --- | --- | --- |
| **N** | **299855** | **318065** | **335771** | **355019** | **369600** | **384826** | **395470** | **402312** | **401175** | **404252** | **400209** | **394266** | **-** |
| **Antithrombotic drugs** |  |  |  |  |  |  |  |  |  |  |  |  |  |
| Antiplatelet, n (%) | 97570 (32.5) | 105216 (33.1) | 114506 (34.1) | 120667 (34.0) | 124006 (33.6) | 121012 (31.4) | 124586 (31.5) | 123688 (30.7) | 121570 (30.3) | 120921 (29.9) | 118370 (29.6) | 115022 (29.2) | <0.001 |
| Anticoagulant, n (%) | 15538 (5.18) | 16377 (5.15) | 18240 (5.43) | 19543 (5.50) | 21224 (5.74) | 20282 (5.27) | 23764 (6.01) | 25271 (6.28) | 25991 (6.48) | 26558 (6.57) | 25902 (6.47) | 24456 (6.20) | <0.001 |
| **Antihypertensive drugs** |  |  |  |  |  |  |  |  |  |  |  |  | <0.001 |
| Calcium antagonist, n (%) | 54209 (18.1) | 58779 (18.5) | 64912 (19.3) | 70619 (19.9) | 74415 (20.1) | 76438 (19.9) | 81858 (20.7) | 84495 (21.0) | 85787 (21.4) | 87567 (21.7) | 88443 (22.1) | 88693 (22.5) | <0.001 |
| Angiotensin receptor blockers (ARB), n (%) | 62710 (20.9) | 69249 (21.8) | 76112 (22.7) | 81520 (23.0) | 84572 (22.9) | 85550 (22.2) | 87916 (22.2) | 88204 (21.9) | 87928 (21.9) | 88683 (21.9) | 88404 (22.1) | 87485 (22.2) | 0.626 |
| Beta blocker, n (%) | 42948 (14.3) | 46003 (14.5) | 51869 (15.4) | 57733 (16.3) | 63005 (17.0) | 66173 (17.2) | 74333 (18.8) | 78962 (19.6) | 82235 (20.5) | 85931 (21.3) | 88145 (22.0) | 89411 (22.7) | <0.001 |
| Angiotensin converting enzyme (ACE) inhibitors, n (%) | 94684 (31.6) | 98629 (31.0) | 106002 (31.6) | 112498 (31.7) | 118034 (31.9) | 121146 (31.5) | 130134 (32.9) | 134267 (33.4) | 135770 (33.8) | 137846 (34.1) | 137981 (34.5) | 138149 (35.0) | <0.001 |
| Diuretic, n (%) | 116025 (38.7) | 123332 (38.8) | 132152 (39.4) | 140691 (39.6) | 148179 (40.1) | 150685 (39.2) | 160830 (40.7) | 165748 (41.2) | 167054 (41.6) | 168952 (41.8) | 168079 (42.0) | 166728 (42.3) | <0.001 |
| Others antihypertensive drugs, n (%) | 11529  (3.8) | 11652  (3.7) | 12212  (3.6) | 11459  (3.2) | 11203  (3.0) | 7318  (1.9) | 6462  (1.6) | 5666  (1.4) | 5082  (1.3) | 4760  (1.2) | 4249  (1.1) | 3829  (0.9) | <0.001 |
| **Lipid-lowering drugs** |  |  |  |  |  |  |  |  |  |  |  |  | <0.001 |
| Statins, n (%) | 113855 (38.0) | 125660 (39.5) | 143733 (42.8) | 160329 (45.2) | 172937 (46.8) | 176881 (46.0) | 189749 (48.0) | 193368 (48.1) | 194412 (48.5) | 195530 (48.4) | 194490 (48.6) | 194051 (49.2) | <0.001 |
| Fibrates, n (%) | 12926  (4.3) | 13864  (4.4) | 15398  (4.6) | 16542  (4.7) | 17328  (4.7) | 17910  (4.7) | 19549  (4.9) | 20280  (5.0) | 21134  (5.3) | 21641  (5.4) | 21992  (5.5) | 22084  (5.6) | <0.001 |
| Ezetrol, n (%) | 3568  (1.2) | 4162  (1.3) | 4619  (1.4) | 4751  (1.3) | 5116  (1.4) | 5327  (1.4) | 5757  (1.5) | 5889  (1.5) | 6556  (1.6) | 7779  (1.9) | 8963  (2.2) | 10429  (2.7) | <0.001 |
| Other lipid-lowering drugs, n (%) | 1954 (0.652) | 2607 (0.820) | 3491 (1.04) | 4332 (1.22) | 4951 (1.34) | 2709 (0.704) | 1816 (0.459) | 2175 (0.541) | 2425 (0.604) | 2085 (0.516) | 2320 (0.580) | 3163 (0.802) | <0.001 |

* Chi-square trend test

Supplementary table 5. Trends in the control of blood pressure, HbA1c, and LDL-cholesterol in patients with and without cardiovascular disease

|  | **2007** | **2008** | **2009** | **2010** | **2011** | **2012** | **2013** | **2014** | **2015** | **2016** | **2017** | **2018** | **p-value*** |
| --- | --- | --- | --- | --- | --- | --- | --- | --- | --- | --- | --- | --- | --- |
| **N** | **299855** | **318065** | **335771** | **355019** | **369600** | **384826** | **395470** | **402312** | **401175** | **404252** | **400209** | **394266** | **-** |
| Patients with HbA1c available, n (%) | 189078  (63.1) | 203420  (64.0) | 219979  (65.5) | 230458  (64.9) | 245642  (66.5) | 267872  (69.6) | 279531  (70.7) | 287588  (71.5) | 294391  (73.4) | 300135  (74.2) | 301135  (75.2) | 303524  (77.0) | <0.001 |
| HbA1c <7 %, n (%) | 103804 (54.9) | 107406 (52.8) | 114829 (52.2) | 123098  (53.4) | 125021 (50.9) | 139401 (52.0) | 158689 (56.8) | 164960 (57.4) | 165211 (56.1) | 167754 (55.9) | 170135 (56.5) | 169508 (55.9) | <0.001 |
| HbA1c <8 %, n (%) | 149372  (79.0) | 158261  (77.8) | 171584  (78.0) | 181350  (78.7) | 190463  (77.5) | 210435  (78.6) | 226872  (81.2) | 234273  (81.5) | 238265  (80.9) | 242467  (80.8) | 244797  (81.3) | 246248  (81.1) | <0.001 |
| Patients with BP available, n (%) | 231947  (77.4) | 244153  (76.8) | 258951  (77.1) | 274844  (77.4) | 287559  (77.8) | 308028  (80.0) | 316365  (80.0) | 324968  (80.8) | 330230  (82.3) | 337059  (83.4) | 331200  (82.8) | 329188  (83.5) | <0.001 |
| Blood pressure, ≤140/90 mmHg, n (%) | 127500 (55.0) | 141370 (57.9) | 157401 (60.8) | 174196 (63.4) | 189809 (66.0) | 218883 (71.1) | 230301 (72.8) | 228088 (70.2) | 234321 (71.0) | 241471 (71.6) | 235144 (71.0) | 236469 (71.8) | <0.001 |
| All Patients with LDL-C available, n (%) | 171437  (57.2) | 187627  (59.0) | 205134  (61.1) | 224593  (63.3) | 233227  (63.1) | 249473  (64.8) | 260784  (65.9) | 263844  (65.6) | 270575  (67.4) | 272986  (67.5) | 273984  (68.5) | 277160  (70.3) | <0.001 |
| All patients LDL-C <100 mg/dL, n (%) | 57100  (33.3) | 68311  (36.5) | 72282  (35.2) | 84110  (37.4) | 96010  (41.2) | 104857  (42.0) | 120659  (46.0) | 124071  (47.0) | 127568  (47.0) | 133306  (48.8) | 132947  (48.5) | 134393  (48.4) | <0.001 |
| LDL <100 mg/dL without CVD, n (%) | 41036  (29.6) | 48173 (32.1) | 49620  (30.6) | 57046  (32.5) | 65132 (35.9) | 69985 (36.6) | 80576 (40.6) | 81957 (41.0) | 83185 (40.8) | 85825 (41.9) | 84776 (41.4) | 84877 (41.2) | <0.001 |
| LDL<100 mg/dL with CVD, n (%) | 16064  (48.8) | 20138  (53.2) | 22662 (52.4) | 27064 (55.2) | 30878 (58.9) | 34872 (60.2) | 40083 (64.6) | 42114 (66.0) | 44383 (66.6) | 47481 (69.1) | 48171 (69.3) | 49516 (69.7) | <0.001 |

CVD: Cardiovascular diseases; LDL-C: low-density lipoproteins; BP: blood pressure; * Chi-square trend test

Supplementary table 6. Trends in the composite indicator of the three cardiovascular risk factors (blood pressure, HbA1c and LDL-cholesterol) in patients with and without cardiovascular disease

|  | **2007** | **2008** | **2009** | **2010** | **2011** | **2012** | **2013** | **2014** | **2015** | **2016** | **2017** | **2018** | **p-value*** |
| --- | --- | --- | --- | --- | --- | --- | --- | --- | --- | --- | --- | --- | --- |
| Patients with available data  for the three CVRF, n (%) | 146264  (48.8) | 159560 (50.2) | 174547 (52.0) | 184741  (52.0) | 194698 (52.7) | 219085  (56.9) | 228302  (57.7) | 232444 (57.8) | 240096  (59.8) | 243629  (60.3) | 242259 (60.5) | 244921  (62.1) | <0.05 |
| Three CVRF  in all patients, n (%) | 18228 (12.5) | 22117 (13.9) | 25515 (14.6) | 25164 (13.6) | 28654 (14.7) | 35805 (16.3) | 46278 (20.3) | 46952 (20.2) | 47248 (19.7) | 49634 (20.4) | 49324 (20.4) | 49211 (20.1) | <0.05 |
| Three CVRF controlled  in patients with CVD, n (%) | 5270 (18.7) | 6607 (20.5) | 8150 (22.0) | 8071 (19.9) | 9055 (20.5) | 11695 (22.8) | 14990 (27.2) | 15705 (27.5) | 16199 (27.0) | 17486 (28.2) | 17752 (28.6) | 17994 (28.3) | <0.05 |
| Three CVRF controlled  in patients without CVD, n (%) | 12958 (11.0) | 15510 (12.2) | 17365 (12.6) | 17093 (11.9) | 19599 (13.0) | 24110 (14.4) | 31288 (18.1) | 31247 (17.8) | 31049 (17.2) | 32148 (17.7) | 31572 (17.5) | 31217 (17.2) | <0.05 |

CVRF: cardiovascular risk factor; Three CVRFs: HbA1c ≤7%, Blood pressure ≤ 140/90 mm Hg, LDL-C< 100 mg/dl ; CVD: Cardiovascular disease; LDL-C: low-density lipoproteins; * Chi-square trend test
